# Preventable hand injuries presenting to a dedicated hand and wrist unit in England: a pilot study

**DOI:** 10.1177/17531934211019297

**Published:** 2021-05-26

**Authors:** Justine Silber, Grey Giddins, Maxim D. Horwitz

**Affiliations:** 1 Birmingham Medical School, University of Birmingham, Edgbaston, UK; 2 Department of Orthopaedic Surgery, Royal United Hospitals NHS Foundation Trust, Bath, UK; 3 Department of Hand Surgery, Chelsea and Westminster Hospital, London, UK

Dear Editor,

The annual incidence of hand injuries in England is estimated to be 110 per 100,000 population (Manley et al., 2019), accounting for a notable proportion of attendances at Accident and Emergency Departments. Many of these may be preventable. Despite health and safety regulations reducing the incidence of agricultural and industrial injuries, preventable injuries in other settings occur with unknown frequency.

We aimed to define the term ‘preventable hand injury’ and, based on this, to undertake a prospective pilot study of consecutive patients with new wrist or hand injuries presenting to a dedicated hand unit in England over a 2-month period, to assess the incidence and context of such injuries, and to outline prevention strategies. This pilot study assesses feasibility and refines methods for a larger national audit of preventable hand injuries.

We defined preventable hand injury as ‘an injury to that hand (e.g. laceration, abrasion) that is innate to the activity being performed and that would not have occurred if reasonable human interventions were in place’. Sports-based injuries are excluded as there is an inherent risk of playing sport, and, in general, the benefits of sport outweigh the risks to the wrist and hand. Alcohol and narcotics-related injuries are also excluded as it is unlikely that anything except extreme measures, such as banning alcohol or driving, could prevent these injuries. Burn injuries could be considered preventable, but they were excluded from this study as management is by separate units.

In September–October 2019, clinicians on site completed a standardized proforma during or at the end of each clinic. Data included demographic information, hand dominance, mechanism and description of injury, and management plan. It is acknowledged that the definition of a preventable injury is open to interpretation, thus, to ensure consistency, injuries were categorized by the same person (JS), with queries resolved with senior clinician MH.

Twenty-eight per cent (136/493) of patients were classified as preventable, of whom 62 % were men. The median age was 32 years (range 9 months to 92 years). Table 1 shows preventable injuries by context of injury, age and sex. Door and window injuries were the most common (31%), accounting for most injuries in 0–10 year olds. Injuries to manual workers are often similar to do-it-yourself injuries, but were separated as professionals should have more training, and guidance on reducing injury in the workplace already exists. In the ‘other’ category, two of these were directly due to lawnmower injuries. The do-it-yourself and manual work injuries were predominantly in men; however, kitchen injuries occurred roughly equally in men and women.

**Table 1.** The context of preventable injuries by sex and age group in 136 patients.

Patient characteristics	Context of injury						
	Do-it-yourself activities	Manual work	Door and window injuries	Kitchen activities	Other	Total	
Sex (men/women)	21/6	19/1	19/23	18/20	7/2	84/52	(62/38%)
Age (years)							
0–10	0	0	10	0	1	11	(8%)
11–20	2	1	3	2	0	8	(6%)
21–30	3	4	10	7	1	25	(18%)
31–40	8	5	6	10	1	30	(22%)
41–50	5	4	4	9	4	26	(19%)
51–60	3	3	5	9	1	21	(15%)
61–70	3	3	1	1	0	8	(6%)
>70	3	0	1	0	1	5	(4%)
Total	27 (20%)	20 (15%)	42 (31%)	38 (28%)	9 (7%)	136	

Data in this table are number of patients (%).

When classified by injury description, the most common injuries were: nail injuries (25%), lacerations (23%), closed fractures (23%) and tendon/ligament injures (9%). Patients often presented with combined injuries. Nearly half of preventable injuries (47%) were treated non-operatively. A similar proportion of patients were treated under local anaesthetic, either in the operating theatre or in the clinic. Ten patients (7%) required surgical procedures under general anaesthetic, almost all of these injuries were tendon or nerve lacerations.

Hand injuries cause notable morbidity and disability to patients. This disproportionately impacts the younger, working population. Furthermore, there are healthcare associated costs, with the cost of each hand injury to the National Health Service ranging from £140 to £5300 (NHS Improvement and NHS England, 2020).

We found that nearly one-third of hand injuries were preventable. This highlights the need to develop strategies to reduce these injuries. Prevention could include educational, legislative and technological approaches, either singularly or in combination. The Cochrane Reviews of injury prevention provide frameworks. For example, the review on injury prevention in agriculture (Rautiainen et al., 2008) uses ‘Three E’s of Safety’: *engineering* or technology changes to the equipment used, such as tool design; *education* and behaviour changes, such as training programmes, public safety posters and warning labels; and *enforcement* or legislative changes, such as government regulation of machinery. A similarly combined prevention approach could be applied to door and window injuries and for food preparation.

This research, and the need for prevention strategies, became even more relevant in the context of the self-isolation and lockdown related to the COVID-19 pandemic, which caused individuals to spend more time at home where many preventable injuries occur. In this context, the recent British Society for Surgery of the Hand (BSSH) (2020) media campaign to raise awareness of preventable hand injuries is an educational intervention that is both necessary and timely. To ensure that behaviour change is sustained, there is a need for ongoing messaging, together with the development and evaluation of multi-faceted interventions.

Further research on the frequency, causes and impact of preventable hand injuries is needed to underpin these interventions and health promotion activities and monitor their impacts. This pilot study provides data showing that a larger national study is necessary and feasible with the dual aims of developing a sharp definition of the term *preventable hand injury* and providing a large dataset to facilitate behavioural change and inform policy and practice.
